# Desserts

**Published:** 1874-11

**Authors:** 


### DESSERTS.

WHAT, in the name of common sense, is the reason that some people have got to think and teach that desserts are rank poison? as if the Beneficent One had not "given us all things richly to enjoy!" Are they wild because they can't afford to have nice pies and delicious puddings, and the good old apple-dumplings of our happy childhood? They are all quite as nutritious as porter-house steak, and certainly more so than geese and gobblers. The water-cure people, and the very peculiar kind of mind which affiliates with them, seem to think, that because a thing is "good to eat" it must be discarded from our tastes. Well-made pastry is as good for a hungry man as roast beef. Pies and puddings are just as good as bread; and yet "bread," the bread which disgraces New England, and takes so generally, as heavy as lead, tough as shoe leather, and as indigestible as a brass button, has made millions of dyspeptics; and so have pies and puddings; but it is because they were "bad," were not well made. If a man is "hurt" because he ate a good pie, it is because he made a fool of himself by eating too much. So far from pies and puddings, and desserts in general, causing dyspepsia, they would help to cure it in all cases, if they are good of their kind, and eaten at a proper time, that is,

EAT THEM YESTERDAY.

Every observant reader has had it in his own experience, that having eaten a good dinner—as much as he wanted—a luscious dessert has been unexpectedly brought in, when he has "turned to" and eaten almost as much of it in amount as of the original dinner—that is, has made a pig of himself. The dessert is no more to blame for any mishap afterwards than the original meal. It is the dessert and the dinner which cause trouble afterwards. The perfect and always practicable remedy is: if you have ordered a splendid dinner for to-morrow, eat the dessert for to-day's dinner.

In some places in France, last year, we noticed a cute modification of this plan. The dessert was placed on the table first, and about ten minutes given to nibble at one kind, then another The result was, pecuniarily to the landlord, the ravenousness of the guest was abated, and there was not near so much food "bolted" as there would otherwise have been; and, physiologically, the stomach, not having been overloaded with a double meal, was not taxed beyond its capabilities, and the dinner had no other pains or penalties.

If any reader has been taking medicine all his life, and is getting no better, just let him try an experiment for a week, especially if his ailment is of a dyspeptic or bilious character. Take half a pound or more of grapes, or half a pint or more of berries, in their ripe, raw, natural, and fresh state, half an hour before he sits down to his regular meal ; take nothing else between meals ; no dessert immediately after, thus taking fruits as a dessert before instead of after the regular meal ; carefully note the results, and thereafter act accordingly; and, if plainly and succinctly reported, it is quite certain that the practical results of disseminating the information will do great good.

